# *In vitro* growth of the ovarian follicle: taking stock of advances in research

**DOI:** 10.5935/1518-0557.20210076

**Published:** 2022

**Authors:** Neda Taghizabet, Soghra Bahmanpour, Nehleh Zarei Fard, Fatemeh Rezaei-Tazangi, Ashraf Hassanpour, Ebrahim Kharazi Nejad, Fereshteh Aliakbari, Farzaneh Dehghani

**Affiliations:** 1 Department of Anatomical Sciences, School of Medicine, Shiraz University of Medical Sciences, Shiraz, Iran; 2 Department of Anatomical Sciences, Faculty of Medicine, Fasa University of Medical Sciences, Fasa, Iran; 3 Abadan School of Medical sciences, Abadan, Iran; 4 Men's health and reproductive health research center, Shahid Beheshti University of Medical Sciences, Tehran, Iran

**Keywords:** follicle culture, follicle, folliculogenesis

## Abstract

Several factors are necessary for the growth and survival of healthy follicles in the folliculogenesis process, including endocrine and paracrine glands, and a regulated ratio of granulosa cells to oocytes. One of the most powerful methods for studying folliculogenesis is the culture of ovarian follicles and oogenesis within a completely controlled environment. Follicle culture systems are highly developed and are rapidly evolving. However, the methods for separating the follicles, the cultivation techniques, the culture medium, and the dietary and hormonal supplements vary depending on the species studied. This study made a literature review of follicular culture techniques, and we investigated the heterogeneity among these key variables in follicular culture.

## INTRODUCTION

**Figure 1:**
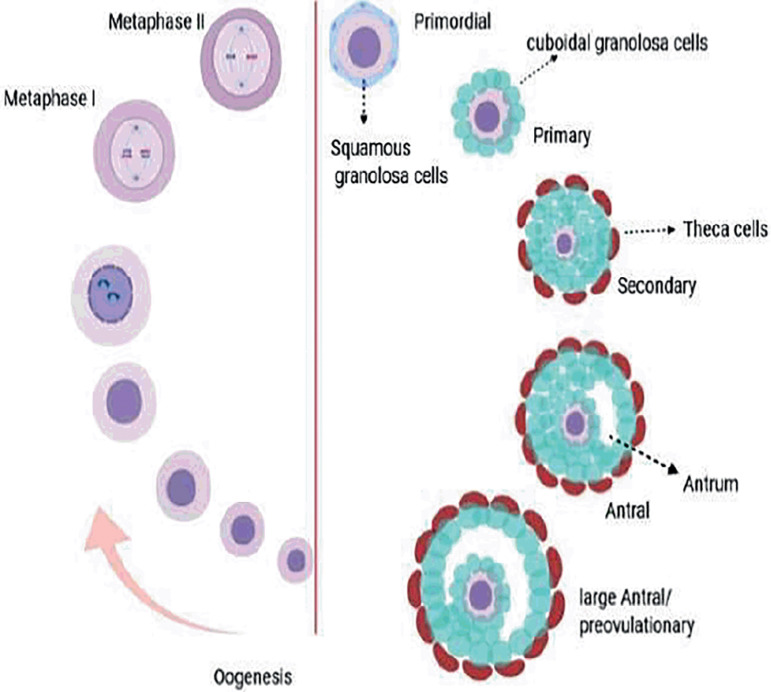
Key steps in developing follicles and oocytes.

The ovaries produce steroid hormones as well as fertilized eggs. The ovarian function unit is the follicle. Each follicle contains one egg surrounded by granulosa and Theca cells (Edson *et al*., 2009). Folliculogenesis starts with the transformation of primordial follicles into primary follicles and the transformation of granulosa cells into cube cells. Granulosa cells proliferate, the oocyte grows, and a secondary follicle takes shape. Theca cells produce androgens. They differentiate outside the basal membrane, and the follicles are dependent on gonadotropins. When a cavity filled with follicular fluid forms, this is called the antral follicle. Depending on the species, folliculogenesis completes one or more follicles and ovulation occurs, but the remaining follicles are involved in the growth process, and suffer from atresia (Mesbah *et al*., 2018; Green & Shikanov, 2016; West *et al*., 2007; Bahmanpour *et al*., 2020). Folliculogenesis and oogenesis are controlled by complicated paracrine, autocrine and juxtacrine genetic factors, and are vital to sustainable fertility (Dehghani *et al*., 2018; Matzuk & Burns, 2012; Richards & Ascoli, 2018) (Figure 1).

A number of *in vitro* follicular culture systems have been developed to preserve the reproductive ability of threatened species or iatrogenic infertility in women (Marin *et al*., 2018). In addition, it is used as a method to identify the toxicity of medications and undesirable fertility chemicals *in vitro* (Xu *et al*., 2015a). There is now a broad spectrum of culture techniques. Here we investigated the follicular culture variables in detail. Including species differences, age, isolation techniques, two-dimensional (2D) *vs*. three-dimensional (3D) systems, cultivation medium and hormonal supplementation.

### Follicle culture systems in different species

Follicle cultures occur in a variety of species. Oocyte growth rate and follicle size (Griffin *et al*., 2006) vary between species (Pepling *et al*., 2010). Follicles are usually classified according to diameter. The term "preantral follicles" is used to describe their different phases (Mehrabianfar *et al*., 2020). Follicles produced *in vitro* are small compared to follicles produced *in vivo* (Xiao *et al*., 2015; Rodrigues *et al*., 2015). Rodents and mammals are the most prevalent models, approximately one-fifth of the studies use human follicles (Xiao *et al*., 2015; Telfer *et al*., 2008), and other mammalian follicles, like the Rhesus monkey (Rodrigues *et al*., 2015; Xu *et al*., 2009a; Peluffo *et al*., 2010; Xu *et al*., 2011a; Hornick *et al*., 2012; Xu *et al*., 2013; Xu *et al*., 2015b; Xu *et al*., 2018; Baba *et al*., 2017); baboon (Xu *et al*., 2011b); bovine (Yamamoto *et al*., 1999; Rossetto *et al*., 2013a;b; Araújo *et al*., 2015); ovine (Arunakumari *et al*., 2010; Muruvi *et al*., 2005); caprine (Rossetto *et al*., 2013a; Ferreira *et al*., 2018; Silva *et al*., 2015; Magalhães *et al*., 2011); swine (Hirao *et al*., 1994; Wu *et al*., 2001); cats (Songsasen *et al*., 2017; Thongkittidilok *et al*., 2018); dogs (Songsasen *et al*., 2011); horses (Haag *et al*., 2013); wildcats (Wiedemann *et al*., 2013).

The main reason for the differences between species is the difference in follicular culture outcomes. For instance, the diameter of the follicles in large mammal species in the preantral stage is much larger than in rodents. In the pre-ovulation stage, adult mouse follicles have a diameter of 420 µm; ovine 600 µm; 750 microns in goats; swine 800 µm and 20 000-23 000 µm in cattle and humans; respectively (Simon *et al*., 2020).

Folliculogenesis and ovulation stages are also different from one species to another. For example, mice follicles reach their maximum diameter within 19 days (Hoage & Cameron, 1976); but large mammals require months (Scaramuzzi *et al*., 2011). Because the growth stages of the follicles in large mammals are long, the presence of nutrients, gas exchange, and hormonal needs are the main challenges of cultivation (Telfer & Zelinski, 2013; West *et al*., 2007). Follicle structures vary from species to species as well. For instance, in large mammals, the theca cellular layer is thicker and affects the exchange of food and gas. Follicle culture and live birth have occurred in mice (Xu *et al*., 2006a;b). However, the follicle culture of rats (Daniel *et al*., 1989), pigs (Wu *et al*., 2001), buffaloes (Manjunatha *et al*., 2007), sheep (Arunakumari *et al*., 2010), goats (Magalhães *et al*., 2011), and Rhesus monkeys (Peluffo *et al*., 2012) were successful in pre-implantation after fertilization. *In vitro* oocyte maturation (IVM) have also been observed in rhesus monkey (Peluffo *et al*., 2010) and Baboon (Xu *et al*., 2011b) follicles. In general, for different reasons, particular species have been used in different follicular culture studies.

### Age and growth stages for follicle culture in different species

In most rodent follicle culture studies, prepubertal follicles have been used, and in less than 30% of adult follicles (Diaz *et al*., 2007; Simon *et al*., 2020). Young animals of reproductive age have been used in studies of mammalian follicles such as sheep (Thomas *et al*., 2003; Arunakumari *et al*., 2010), goats (Ferreira *et al*., 2018; Magalhães *et al*., 2011), and cattle (Gutierrez *et al*., 2000; Itoh *et al*., 2002; Araújo *et al*., 2014a;b; 2015). Prepubertal follicles and smaller follicles have been used to evaluate the use of FSH supplementation in cattle and sheep (Wandji *et al*., 1996; Cecconi *et al*., 1999; Muruvi *et al*., 2005). Prepubertal follicles were used in comparison to the follicles of young and adult goats in 2D or 3D culture system (Leal *et al*., 2018). Prepubertal follicles were used to assess whether smaller preantral follicles could develop into antral follicles *in vitro* (Wu *et al*., 2001). In dogs, different stages of the estrus (Songsasen *et al*., 2011), and in marsupials (Nation & Selwood, 2009) were used in follicular cultures. In the rhesus monkey, the follicles used were primarily of young animals of reproductive age (Rodrigues *et al*., 2015; Baba *et al*., 2017; Xu *et al*., 2018). Small adult follicles were cultivated in adult baboons and were capable of producing live embryos (Xu *et al*., 2011b). Follicular culture studies have been conducted on different species at different ages and cycle stages; and demonstrate that these factors are chosen based on study objectives and ease of access to ovarian tissue.

### Procedures for isolating the follicle

The separation of the follicle from the ovary tissue is the first step in follicle cultivation. Isolated follicles should have a similar morphology (Demeestere *et al*., 2002). Generally, the techniques of separating the follicles from the ovarian tissue include enzymatic, mechanical, or both. In the enzymatic separation of the follicles, proteolytic extracellular matrix (ECM) digestion such as collagenase, deoxyribonucleic, or liberase is utilized. The number of follicles obtained is typically higher in the enzyme digestion method and in compared to mechanical separation methods, they require less time, particularly for fibrous tissues in house mammals (Araújo *et al*., 2014a;b). However, in the enzyme digestion method, the follicles are more likely to be damaged. In mice, for example, collagenase leads to the production of preantral granulosa cell-oocyte complexes (PGOCs) and cell-oocyte complexes (COCs) from ovarian tissues, rather than whole follicles. In the mechanical separation method, special needles are used to separate the follicles of the ovarian stroma or tissue grinders, homogenizers, and cell strainers (Songsasen *et al*., 2017; Mahalingam *et al*., 2016a;b; Craig *et al*., 2010). The mechanical separation method results in less damage to the follicle than the enzyme method, and provides improved protection to the theca layer and follicular morphology (Araújo *et al*., 2014a;b), but the worst problem is that this method takes a lot of time (Demeestere *et al*., 2002). Usually, the selection of the isolating method depends on the follicular stage and the species used in the study. Generally, a short enzymatic digestion step and mechanical separation are used to maintain the structure of the follicle and obtain the maximum number of follicles (Table 1).

**Table 1.** Summary of follicular isolation methods in follicular culture studies.

Isolation	Species	Follicle - Stage	References
Enzymatic	Bovine	Preantral (60–179µm)	Wandji *et al*., 1996
	Canine	Pre-and early antral (100–500µm)	Songsasen *et al*., 2011
	Human	Preantral (90–240µm)	Yuan & Guidice, 1999
		Immature and secondary (176.46±7.20µm)	Laronda *et al*., 2014
		Class I and II (90µm and <90µm)	Roy & Treacy, 1993
		Primordial/primary follicle (≤60µm) Primary/early secondary follicle (>60–120µm) Secondary (>120–250µm)	Yin *et al*., 2016
		Small preantral follicles (42.98±9.06µm)	Amorim *et al*., 2009
	Murine	PGOC	Eppig, 1991 Diaz *et al*., 2007 O’Brien *et al*., 2003
		PGOC and COC	Sugiura *et al*., 2010 Pangas *et al*., 2003
		Secondary (100–130µm)	Desai *et al*., 2012
		Small follicles	Torrance *et al*., 1989
	Ovine	Primordial and primary (40–60µm)	Muruvi *et al*., 2005
Mechanical	Bovine	Preantral (≥190µm)	Araújo *et al*., 2015
		Preantral (166±2.15µm)	Gutierrez *et al*., 2000
		Preantral (190.0±6.6µm)	Araújo *et al*., 2014
		Secondary (268.6±4.5µm)	Antonino *et al*., 2019
		Preantral (145–170µm)	Itoh *et al*., 2002
		Secondary (≥150µm)	Rossetto *et al*., 2013a
	Caprine	Secondary (≥150µm)	Rossetto *et al*., 2013a
		Preantral (>200µm)	Magalhães *et al*., 2011
		Preantral and early antral (~250µm, ~350µm)	Ferreira *et al*., 2016
		Preantral (150–250µm)	Silva *et al*., 2015
	Human	Secondary (≥100µm)	McLaughlin *et al*., 2014
		Secondary (100–150µm)	McLaughlin *et al*., 2018
		Preantral (66–132µm)	Telfer *et al*., 2008
		Multi-layered secondary (165.8±32.3µm)	Xiao *et al*., 2015
		Preantral (>120µm)	Abir *et al*., 1997
	Marsupial	Primordial (63.6–215.5µm)	Nation & Selwood, 2009
	Murine	Secondary (111–137µm)	Jin *et al*., 2010
		Preantral (85–115µm)	Hornick *et al*., 2013
		Two-layered: (100–130µm); multi-layered: (150–180µm)	Kreeger *et al*., 2005; 2006
		Two-layered secondary (100–130µm)	Shikanov *et al*., 2009 Xu *et al*., 2006b Mainigi *et al*., 2011
		Primary (60–80mm); two-layered (90–100µm)	Tagler *et al*., 2014
		Secondary (~90, 100–105, or 120µm)	Tingen *et al*., 2011
		Secondary (180–210µm)	Skory *et al*., 2015
		COC	Buccione *et al*., 1990
		Antral (360.94±16.1µm)	Craig *et al*., 2010
		Antral (200–350µm)	Craig *et al*., 2013
		Antral (250–400µm)	Hannon *et al*., 2015 Hannon *et al*., 2015 Peretz & Flaws, 2013 Zhou & Flaws, 2017 Patel *et al*., 2016 Peretz *et al*., 2013
		Antral (225–400µm)	Mahalingam *et al*., 2016a; 2016b
		Antral (>200 µm)	Miller *et al*., 2005
		Preantral (180–240µm)	Wycherley *et al*., 2004
		Preantral (150–200µm)	Adam *et al*., 2004
		PGOC	Eppig, 1980
		Early preantral (100 and 130µm)	Adriaens *et al*., 2004
		Preantral (150-160µm)	Heise *et al*., 2005
		Preantral (140-170µm)	Heise *et al*., 2009
	Ovine	Preantral small (130±10µm) Preantral medium (185±14µm) Preantral large (250±10µm)	Cecconi *et al*., 1999
		Preantral (161±2µm)	Thomas *et al*., 2003
		Preantral (250–400µm)	Arunakumari *et al*., 2010
	Porcine	Preantral (296±9µm)	Wu *et al*., 2001
	Rhesus	Secondary (100–300µm)	Xu *et al*., 2009a
		COC	Peluffo *et al*., 2012
		Small antral (≥0.5mm)	Peluffo *et al*., 2013
		Secondary (140–225µm)	Xu *et al*., 2018
		Secondary (125–250µm)	Baba *et al*., 2017
		Secondary (125–225µm)	Rodrigues *et al*., 2015
		Secondary (125–250µm)	Ting *et al*., 2015
		Primary (80–120µm) secondary (125–225µm)	Xu *et al*., 2013
		Secondary (130–220µm)	Xu *et al*., 2015b
	Feline	Secondary (100–200µm)	Songsasen *et al*., 2017
		Secondary (208±7.9µm diameter) Early antral (329.8±5.4µm)	Thongkittidilok *et al*., 2018
Combined Enzymatic/Mechanical	Baboon	Preantral (270–300µm)	Xu et al., 2011b
	Human	Secondary (74–260µm)	Skory *et al*., 2015
		Primary (47.0±8.2µm)	Abir *et al*., 1999
		Preantral (190±30µm)	Aziz *et al*., 2017
		Secondary (~170µm)	Xu *et al*., 2009b
	Murine	Preantral (~60–69µm)	Oktem & Oktay, 2007
		Preantral follicles and COC	Vanderhyden *et al*., 1992
		Immature secondary (140–150µm)	Shikanov *et al*., 2011
		Multi-layered secondary (150–180µm)	Xu *et al*., 2006a
	Porcine	Preantral (200–300µm)	Hirao *et al*., 1994
	Rhesus	COC	Peluffo *et al*., 2010
		Secondary (125–225µm)	Xu *et al*., 2011a Xu *et al*., 2010

### Culture systems

**Figure 2:**
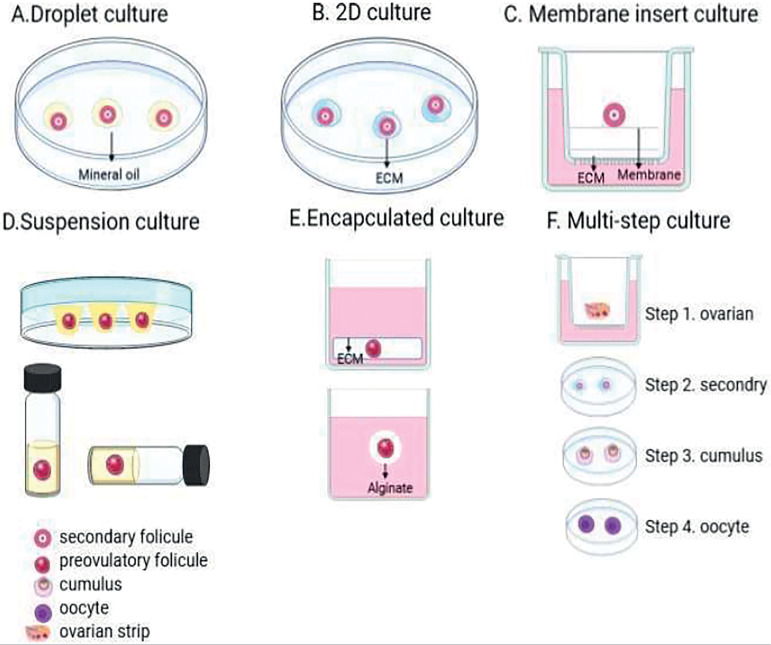
Schematic overview of 2D, 3D and multi-stage culture systems.

Follicular culture systems are known as two-dimensional (2D) or three-dimensional (3D) (Figure 2). In 2D cultures, the follicles are static, but in 3D cultures, the follicles float in biomaterial matter (West *et al*., 2007). 2D-systems include the droplet method, substrate method (ECM coating), and membrane insert systems. In general, the 2D-method is used for small culturing follicles, hormonal studies, and gene expression studies. It is difficult to evaluate folliculogenesis and oocyte maturation in the 2D-method, because during oocyte proliferation, granulosa cells migrate to the surface of the culture medium (Kreeger *et al*., 2006). Logout of the communication between follicular cells stops follicular growth, inhibits ovulation, and meiosis in the egg (Green & Shikanov, 2016; West *et al*., 2007). In general, the follicles may be maintained for a short period of time in the 2D-culture.

### A - 2D-culture systems

#### 1. Droplet culture

Within the droplet system, each follicle is implanted into a drop of culture medium, and each drop is covered with oil. There are drop methods for different stages and different species including mice (Adam *et al*., 2004; Wycherley *et al*., 2004; Adriaens *et al*., 2004), Rhesus monkey (Peluffo *et al*., 2012), sheep (Arunakumari *et al*., 2010), marsupial (Nation & Selwood, 2009), goat (Rossetto *et al*., 2013a; Ferreira *et al*., 2018) and cows (Araújo *et al*., 2014a;b) have been used. It typically takes about 6-18 days for the droplet method (Nation & Selwood, 2009; Arunakumari *et al*., 2010), A18 (Ferreira *et al*., 2018; Magalhães *et al*., 2011) and 32 (Araújo *et al*., 2014a;b) (Figure 2-A).

#### 2 - 2D-culture

In the two-dimensional method, the follicles are grown directly on a surface covered by ECM compounds, such as collagen, laminin, or Matrigel. ECM plays an important role in folliculogenesis and affects cellular behavior, differentiation, and secretory activity (Desai *et al*., 2010). Collagen compounds have elasticity properties and contribute to intercellular communication, while Matrigel promotes cell proliferation and differentiation (Belli *et al*., 2012). Larger follicles such as preantral and antral follicles, PGOCs, and COCs have been used more in systems with 2D plastic substrates (Zhou & Flaws, 2017; Xu *et al*., 2018; Araújo *et al*., 2015; Patel *et al*., 2016; Mahalingam *et al*., 2016a;b; Peluffo *et al*., 2010). In large mammals, the follicles are larger and require more time in the culture environment to grow. Thus, the growing time of larger follicles may be reduced (Araújo *et al*., 2014a;b), and the duration of the culture varies in hours and days. For example, some studies have used the method to grow mammalian follicles such as those of Rhesus and cattle (Xu *et al*., 2018; Gutierrez *et al*., 2000). A fibronectin-coated plate was also used to culture the primordial and primary follicles of sheep. The growth of follicles was not much different from the follicles cultured in fibronectin-free plates (Muruvi *et al*., 2005) (Figure 2B).

#### 3 - Membrane insert culture

Membrane insertion systems function in the same way as 2D-systems, and may contain ECM protein coatings, but in this method, the follicles are in an insert within a well of a culture plate and immersed in the environment. The mice follicles were cultured using a membrane inserting system, which improved the growth and ovulation of the follicles (Adam *et al*., 2004). For the first time, human follicles were cultured with a membrane insert system for 4 weeks. COC culture studies using membranes coated with ECM proteins (Sugiura *et al*., 2010) were also reported. Other 2D methods of follicle culture, include the use of glass coverslips coated with various ECM components. Although the earliest methods for cultivating ovarian follicles are 2D-systems, the 2D-methods damages the structure of the follicles, so that it is better suited for short-term cultures and small follicles (Figure 2C).

### B - 3D-culture systems

3D-culture acts as *in vivo* and is adapted to long-term follicle culture. A major disadvantage of two-dimensional systems is that it damages the structure of the follicle surrounded by granulosa cells. This system is problematic for the culture of large mammalian follicles, which require culture and long-term communication among cells. In a 3D culture system, the structure of the intact follicles retains, in which the follicles are surrounded by biomaterials or have little access to a substrate. There are different types of 3D-systems, some using different scaffolding and encapsulation follicles, others using floating culture, or using in situ culture. To encapsulate the follicles, several matrices are used, which, in vivo, creates a very restricted environment, similar to that of the ovary and maintains the follicular structure and intercellular communication (Belli *et al*., 2012). Matrix compounds include natural substances such as collagen, alginate, or matrigel, or synthetic compounds such as polyethylene glycol (PEG) hydrogels that bind to protein-sensitive peptides (Figure 2).

#### 1. Suspension culture

In this 3D-system, there is no scaffold and the structure of the follicles is protected by a system of rolls, inversion, or magnetic grains (Nation & Selwood, 2009; Wycherley *et al*., 2004). In marsupials, using inverted droplets, mature oocytes were obtained, which were more effective than follicles cultivated in different systems such as vertical droplets and roller systems. In tubes containing polypropylene, rat follicles produced eggs capable of performing meiosis, and were fertilized with intra cytoplasmic sperm injection (ICSI). Using a 3D magnetic system, cattle follicles produced live eggs that resumed meiosis after *in vitro* maturation (IVM) (Antonino *et al*., 2019) and follicle survival was higher than in the 2D-system (Figure 2D).

#### 2. Encapsulated culture

In these culture systems, a biocompatible substance such as agar and collagen surround the follicle and protects its 3D structure. These materials are placed in layers on culture sheets to insert the follicles between these layers. In the first report of using the collagen gel matrix in the three-dimensional method, due to the stiffness of the matrix, no antrum was formed. Other studies have used collagen and agar matrices to grow follicles in mice (Vanderhyden *et al*., 1992) and pigs (Hirao *et al*., 1994), which, in comparison to 2D-systems, has maintained follicle structure and extended culture. In human studies, the use of collagen and agar in the 3D system made it possible to maintain the structure of the follicle and the egg for only 24 to 120 hours.

Brown algae are capable of producing a hydrogel called alginate that is biocompatible and can be used as a matrix in follicle culture (Belli *et al*., 2012). Alginate was first used in the culture of mice COCs. The results showed that alginate maintains intercellular communication, the proliferation of granulosa cells, and increases egg volume. Usually, ovarian cortex follicles move from the hard medulla to softer layers as they develop. Results of studies have demonstrated that concentrated alginate contributes to the growth of mice primary follicles, but it is not suitable for the development of larger follicles and the formation of antrum (Xu *et al*., 2006a;b; Skory *et al*., 2015). Also, studies of follicular culture in a 3D-system containing alginate have shown that low levels of alginate contribute to follicular growth, but, concentrated alginate is appropriate for hormone production (Songsasen *et al*., 2011).

Alginate encapsulation was used in other mammals, such as the Rhesus monkey, which could produce embryos at the cleavage stage (Xu *et al*., 2011a). By culturing the follicles in the combination system, the first mature human metaphase II (MII) oocytes were produced. First, the preantral follicles were cultured in 0.5% alginate for 10-15 days, and then the antral follicles were placed in low attachment plates for up to 40 days (Xiao *et al*., 2015). Supplements can impact 2D and 3D-culture systems. For example, one study found that vascular endothelium growth factor (VEGF) contributes to the growth of bovine secondary follicles in the 2D-system, and the growth hormone (GH) induces estradiol production in the 3D-alginate system (Araújo *et al*., 2014a;b). In a study using the caprine model, the encapsulation of 3D alginate was compared to the 2D substrate system that increased follicular survival and increased the number of eggs appropriate for IVM and IVF in the 3D-system. But in the 2D-culture, the follicles produced higher levels of progesterone.

Using the combination of alginate and fibrin, a dynamic permeable fibrin-alginate (IFN) network was developed (Shikanov *et al*., 2009). Within this matrix, follicular proteases degrade fibrin, reducing alginate concentration and matrix rigidity. This matrix mimics the internal environment of the ovary, as in ovarian tissue, follicles smaller than the hard cortex move into the soft marrow (Shikanov *et al*., 2011). With IFN in rodents, high meiotic follicles were developed (Jin *et al*., 2010) but in monkeys, it did not increase secondary follicle production. Embryonic stromal cells and fibroblasts (MEF) in mice were also grown with alginate-encapsulated follicles (Tagler *et al*., 2014). Ovarian stromal cells are involved in the growth, survival, and production of androgens in primary and secondary mice follicles. Culture of MEF cells with primary follicles containing alginate enhanced growth but decreased cell survival. Matrigel matrix is also used in three-dimensional culture, which in addition to maintaining the structure of the follicle, creates a protein-rich environment for folliculogenesis. 

In matrigel, with fibrin and alginate, baboon follicles were enclosed, grew, and were able to produce mature eggs. The hyaluronan matrix was also used to grow follicles (Belli *et al*., 2012). The hyaluronan-ECM (no alginate) matrix on rat follicles increased follicular survival and increased the steroid hormone (Desai *et al*., 2012). The synthetic matrix of polyethylene glycol (PEG) acts like fibrin and is degraded by follicular proteases. Using the PEG matrix increased follicle growth in mouse models by 17 times (Shikanov *et al*., 2011) (Figure 2E).

#### 3. Multi-step culture

Multi-step methods have been developed for follicle growth and the creation of a more similar physiological environment that primordial, primary, and early-secondary stage follicles can be cultured. First, the small follicles are grown in situ in the ovarian natural environment, and then the cultured follicles are separated from this tissue (McLaughlin *et al*., 2014; Jin *et al*., 2010; McLaughlin *et al*., 2018; Telfer *et al*., 2008). This method helps grow human follicles until they become mature gametes. For example, in one study using human ovarian tissue, secondary follicles were isolated and encapsulated in alginate. As the follicles grew and the antrum formed, they were released from the alginate matrix and transferred to low attachment plates for 40 days. Which turned human follicles into mature eggs (Xiao *et al*., 2015). In the next study, an alternative multistage method was used. In the first stage, cortical strips were cultured for 8 days. Secondly, the follicles were cultured for 8 days, and the COC cells were cultured on the membranes for 4 more days (Step 3). In the fourth stage, eggs larger than 100 µm were selected for IVM (McLaughlin *et al*., 2018). Also, a multi-step method was used for follicle growth in rodents. Generally, these systems have been very useful for long-term cultures of large mammal follicles. Therefore, the introduction of microfluidic systems or other natural scaffolding can be very useful in healthy *in vitro* follicles (Gargus *et al*., 2020) (Figure 2F).

### Media composition and supplements

To grow the follicles, it is necessary to enrich the growing medium with nutrients, growth supplements, and hormone compounds. The selected culture medium should protect the growth of follicles and the maturation of eggs. As a result, the main media used in follicular culture typically include minimal essential medium (MEM), Dulbecco's modified eagle medium (DMEM), Waymouth's medium, McCoy's 5a medium), balanced salt solutions (Earle's balanced salt solution) (EBSS)), or mixed media (DMEM + F12, α-MEM + Glutamax).

Also, supplements are added to the follicular culture medium. For example, glucose as a source of carbon energy (Nation & Selwood, 2009), L-glutamine or fetuin as a source of amino acids (Asadi *et al*., 2017); ascorbic acid for reducing apoptosis and maintaining follicular structure, penicillin, streptomycin, and kanamycin as antibiotics (Demeestere *et al*., 2005) is used. Additionally, for the growth of follicles *in vitro* from the combination of ITS (insulin, transferrin, selenium) to increase the absorption of amino acids (Abedelahi *et al*., 2008). Protein supplements such as fetal calf serum (FCS), fetal bovine serum (FBS), and bovine serum albumin (BSA) are used in culture medium. Results from a mice model study showed that over a 10-day period, α-MEM, DMEM, and DMEM + F12 media had a better effect on antrum formation, follicle growth than Waymouth, M199, IMDM, and RPM1640. Also resulted in an increase in the number of MII oocytes (Simon *et al*., 2020). For culturing the human ovarian cortical tissue over a 10-day period, the MEM medium enriched with 10% human serum and 300 mIU/mL FSH may have a greater effect on follicular growth than the Waymouth and EBSS media (Wright *et al*., 1999). In another study, TCM-199 enriched with 10 ng/ml EGF was used over a 7-day period and had a better effect on the growth of goat and sheep follicles than α-MEM with the EMF (Andrade *et al*., 2014).

The TCM199 medium also increased the rate of antrum formation from bovine preantral follicles, relative to a-MEM or McCoy 5a medium (Rossetto *et al*., 2013a;b). Another factor affecting folliculogenesis is oxygen stress. Oxygen 5% is near the physiological oxygen levels. High oxygen stresses may produce reactive oxygen radicals (ROS) with cytotoxic effects (Rajabi *et al*., 2018). In one study, oxygen stress was induced in the follicle culture environment in rats. Which resulted in the production of mature eggs with higher performance in terms of static control. The rate of antrum formation in culture with 5% oxygen from caprine, ovine, and bovine (Gigli *et al*., 2006) follicles had more than 20% oxygen. Also, the culture of dog COCs in 5% oxygen decreased cell apoptosis compared to that in 20% oxygen (Silva *et al*., 2009). Low-oxygen stress along with high FSH and high fetuin in rhesus monkey, increased follicle growth, and antrum formation (Xu *et al*., 2011a). In general, these studies show that the selection of a suitable culture medium for follicle growth depends on the species. Furthermore, the protective effect of oxygen is much more important at the physiological level (Table 2).

**Table 2.** Media usage through various species and follicular stages.

Culture Medium	Species	Follicle Stage	References
Whitten’s medium	Murine	PGOC	Eppig, 1980
Bicarbonate buffered M199	Murine	Small follicles	Torrance *et al*., 1989
Waymouth’s medium	Murine	PGOC	Eppig, 1991 O’Brien *et al*., 2003
	Human	Immature	Laronda *et al*., 2014
	Ovine	Primordial and primary (40–60µm)	Muruvi *et al*., 2005
	Porcine	Preantral (200–300µm)	Hirao *et al*., 1994
Way/IBMX/lTS/BSA medium	Bovine	Preantral (60 to 179µm)	Wandji *et al*., 1996
	Murine	Preantral and COC	Vanderhyden *et al*., 1992
DMEM	Human	Class 1 (90µm) Class 2 (<90µm)	Roy & Treacy, 1993
		Preantral (90–240µm)	Yuan & Guidice, 1999
	Marsupial	Primordial and primary (63.6–215.5µm)	Nation & Selwood, 2009
αMEM	Baboon	Preantral (270–300µm)	Xu *et al*., 2011b
	Bovine	Preantral (190.0±6.6µm)	Araújo *et al*., 2014
		Secondary (≥150µm)	Rossetto *et al*., 2013a
	Caprine	Secondary (≥150µm)	Rossetto *et al*., 2013a
		Secondary (≥150µm)	Rossetto *et al*., 2013a
		Preantral (≥200µm)	Magalhães et al., 2011
		Preantral (150–250µm)	Silva *et al*., 2015
		Preantral (~250µm) early antral (~350µm)	Ferreira *et al*., 2018
	Canine	Pre- and early antral (100–500µm)	Songsasen *et al*., 2011
	Feline	Secondary (208±7.9µm) Early antral (329.8±5.4µm)	Songsasen *et al*., 2017
		Secondary (100-200µm)	Thongkittidilok *et al*., 2018
	Human	Pre- and early antral (≥120µm)	Abir *et al*., 1997
		Secondary (170–178µm)	Xu *et al*., 2009b
		Secondary (176.46±7.20µm)	Laronda *et al*., 2014
	Murine	COC	Pangas *et al*., 2003
		Preantral (150–200µm)	Adam *et al*., 2004
		Preantral (180–240µm)	Wycherley *et al*., 2004
		Antral (≥200 µm)	Miller *et al*., 2005
		Two-layered (100–130µm) Multi-layered (150–180µm)	Kreeger *et al*., 2005; 2006
		Two-layered (100–130µm)	Xu *et al*., 2006b Shikanov *et al*., 2009 Desai *et al*., 2012
		Multi-layered secondary (150–180µm)	Xu *et al*., 2006a
		Preantral (~60–69µm)	Oktem & Oktay, 2007
		Secondary (111–137µm)	Jin *et al*., 2010
		Antral (360.94±16.1µm)	Craig *et al*., 2010
		Immature secondary (140–150µm)	Shikanov *et al*., 2011
		Secondary (~90, 100–105, or 120µm)	Tingen *et al*., 2011
		Early preantral (100–130µm)	Adriaens *et al*., 2004
		Antral (200–350µm)	Craig *et al*., 2013
		Antral (250–400µm)	Peretz & Flaws, 2013 Peretz *et al*., 2013 Hannon *et al*., 2015 Zhou & Flaws, 2017 Patel *et al*., 2016
		Preantral (85–115µm)	Hornick *et al*., 2013
		Primary (60–80 µm) two-layered (90–100µm) Antral (225–400µm)	Tagler *et al*., 2014 Mahalingam *et al*., 2016a; 2016b
		Secondary (180–210µm)	Skory *et al*., 2015
		Preantral (150-160µm)	Heise *et al*., 2005
		Preantral (140-170µm)	Heise *et al*., 2009
		Early secondary (100-130µm)	Mainigi *et al*., 2011
	Ovine	Preantral (small 130±10µm) Preantral (medium 185±14µm) Preantral large (250±10µm)	Cecconi *et al*., 1999
	Rhesus	Secondary (100–300µm)	Xu *et al*., 2009a
		Secondary (125–225µm)	Xu *et al*., 2011a Xu *et al*., 2010 Rodrigues *et al*., 2015
		Primary (80–120µm) secondary (125–225µm)	Xu *et al*., 2013
		Small antral (≥0.5mm)	Peluffo *et al*., 2013
		Secondary (125–250µm)	Ting *et al*., 2015 Baba *et al*., 2017
		Secondary (130–220µm)	Xu *et al*., 2015b
		Secondary (140–225µm)	Xu *et al*., 2018
αMEM + F12	Human	Multi-layered (165.8±32.3µm)	Xiao *et al*., 2015
αMEM + Glutamax	Human	Preantral (190±30µm)	Aziz *et al*., 2017
		Secondary (74–260µm)	Skory *et al*., 2015
		Small preantral (42.98±9.06µm)	Amorim *et al*., 2009
αMEM + TCM199	Bovine	Preantral (≥190µm)	Araújo *et al*., 2015
αMEM + Earle’s balanced salts	Murine	COC and PGOC	Buccione *et al*., 1990 Diaz *et al*., 2007 Sugiura *et al*., 2010
TCM199B	Bovine	Preantral (40–70µm)	Schotanus *et al*., 1997
		Preantral (145–170µm)	Itoh *et al*., 2002
		Secondary (268.6±4.5µm)	Antonino *et al*., 2019
	Ovine	Preantral (250–400µm)	Arunakumari *et al*., 2010
Earle’s Balanced Salts	Human	Primary (47.0±8.2µm)	Abir *et al*., 1999
McCoy’s 5a	Bovine	Preantral (166±2.15µm)	Gutierrez *et al*., 2000
	Human	Preantral (66 to 132µm)	Telfer *et al*., 2008
		Secondary (≥100µm)	McLaughlin *et al*., 2014
		Primordial/primary follicle (≤60µm) primary/early secondary follicle (>60-120µm) Secondary (>120–250µm)	Yin *et al*., 2016
		Secondary (100–150µm)	McLaughlin *et al*., 2018
	Ovine	Preantral (161±2µm)	Thomas *et al*., 2003
NUSC-23 Media	Porcine	Preantral (296±9µm)	Wu *et al*., 2001
TALP	Rhesus	COC	Peluffo *et al*., 2010; 2012

## CONCLUSIONS

The general process of follicular culture has changed a lot from the past until now, and the main purpose of these changes has been to imitate the natural ovarian environment. By identifying the structure of the ovarian scaffold, information about 3D printing of the ovary was obtained. Ovarian function was thoroughly investigated by making 3D-printed scaffolds (Laronda *et al*., 2017). In addition, depending on physiological needs of the cell, other technologies such as microfluidics can be used to grow follicles. In static models, the use of a microfluidic system can be very effective. Because in addition to oxygenation, nutrient exchange and cellular communication, it provides a three-dimensional environment for the follicles (Desai *et al*., 2010). In order to reconstruct the human ovary environment in vitro, factoring plays a major role in the menstrual cycle. Therefore, in the context of a microfluidic chip (Scaramuzzi *et al*., 2011), alginate encapsulation (Gomes *et al*., 2015) was used to mimic the hormonal changes of the menstrual cycle in follicle culture. Microfluidics have made possible the successfully recombine the 28-day human menstrual cycle by fusion of tissues, such as mice ovaries and human fallopian tubes, ectopic uterus, and liver (Xiao *et al*., 2017). Microfluidic operating systems should be readily available and promote follicular culture among different species. Follicle culture methods vary depending on the species, the age of the animals, and the stage of the follicle.
